# Porcelain Corners Retained by Porcelain Pins

**Published:** 1904-02

**Authors:** John M. Byers

**Affiliations:** Brooklyn, N. Y.


### PORCELAIN CORNERS RTEAINED BY PORCELAIN PINS.

By John M. Byers, Brooklyn, N. Y.

Read before the Second District Dental Society.

Probably in no other position in the mouth do the æsthetic qualities of porcelain fillings appear to such advantage as when used to restore the contour of an incisor; and in no other class of porcelain fillings do we meet with more difficulties of retention. The shallow depth to which the matrix can be forced limits the distance to which we can carry our porcelain body. So in proportion to the depth do we have weakness or strength. Therefore, it is good reasoning to assume, that if we can get a deep, solid mass of porcelain in the form of a pin, or block, within the tooth—this pin to be part of the filling and homogeneous with it—we will have a filling that will hold, and withstand more than the ordinary stress of mastication—provided it is perfectly made and properly articulated. Let me add that no such filling should ever be allowed to come in actua contact with the occluding tooth.

I, think you will agree with me that a solid porcelain pin, of sufficient thickness, has greater strength than any *p atinum* pin or bar that can be baked in. With this idea in mind let us proceed to make a corner filling—with a big, thick porcelain pin—that will hold it in place against all the heavy stran to which its exposed position subjects it. We will assume that an upper central incisor presents with a broken corner involving one-quarter or more of ts labial face.

Having obtained a good separation, apply the rubber dam to at least three teeth, obtaining plenty of room to work the matrix. I am aware that some operators do not think it necessary to use rubber dam for porcelain work; but the saving of time, inasmuch as you are not obliged to continually dry the cavity, will aloneb be reason enough, to say nothing of the fact that cement must have considerabe time to dry before exposure to the saliva.

First take a medium garnet paper disk, and cut the edges down until they are flat and true, making a sharp angle with the labial and lingual faces of the enamed. All bevel edges must be sacredly avoided; a flat, even surface is the very foundation of our filling. We will now deepen the interior cavity; take a new bur and cut into the dentine, being careful not to overheat the tooth, and carry the excavation to its greatest possible depth. Cut on perfectly true, straight lines, so that the cavity when completed may present without the faintest suspicion of an undercut. If you will be careful not to overheat by the rapidly revolving bur, and apply a drop of pure carbolic occasionally, you can deepen the cavity to a surprising degree. Always use a new sharp bur.

We will now take a small sheet of platinum and burnish it into the cavity in the usual way. We find that the platinum can be carried in a short distance only, when we come to a point where, if we burnished any further, we would puncture the matrix. Do not go any deeper, but otherwise complete the matrix, carrying it well over the edges, and be sure there is no rocking.

Return now to the interior. We have forced the platinum foil as far as it will go; take a ball burnisher and push it right through the foil into the cavity beneath. Break right in and dress the foil up against the cavity walls. This is the primary matrix. Being satisfied that the adaptation is perfect, carefully remove and lay it aside, and take up the construction of the secondary matrix. Cut an orange wood stick to fit the cavity as perfectly as possible, smoothing it with a fine disk, and if made with care it will serve as an excellent model upon which to mold the secondary matrix. Fold the platinum about the end of the wooden model, turning in the ends, very much as you would do up a paper package. Loosen this platinum cap and carry it to place in the cavity; it will be found to fit fairly well, and the burnisher will complete the adaptation. Allow a slight overlapping at the opening.

Being properly adapted, remove, fill with porcelain body and bake. Fuse a very small portion of body at the bottom first, and gradually build it up, stopping a little short of the opening. When this baking is complete, we have, what in the finished filling will be the pin. The primary matrix must now be replaced, then take the secondary, containing the porcelain pin and carry it into place, forcing it through the primary until it fits properly. Burnish the narrow edges of the secondary matrix down to act as a flange.

Both may now be removed together using special care, and proceed to build up the contour and bake in the usual way. When completed and the platinum stripped off you will have a porcelain corner, reinforced by a strong serviceable pin, and it should fill the cavity so perfectly that only the thinnest cement can be used for the setting. For this class of filling I like to use platinum for my matrix, with the Jenkins body. The first button on a Hammond furnace will give all the heat necessary for fusing. It may be of some assistance to invest the secondary matrix, although it can be handled without. However, after the filling is well under way, I prefer to work without investment, so that the piece may, from time to time, be put into position in the cavity, to inspect the edges and verify the contour. These fillings may be made quickly, but we should give to each individual case all the thought and time necessary; and thereby give better service to our patients, and enhance our own reputations as operators.

